# Genetic Variation in Flowering Traits of Tasmanian *Leptospermum scoparium* and Association with Provenance Home Site Climatic Factors

**DOI:** 10.3390/plants11081029

**Published:** 2022-04-10

**Authors:** Christopher N. Wellington, René E. Vaillancourt, Brad M. Potts, Dale Worledge, Anthony P. O’Grady

**Affiliations:** 1 School of Biological Sciences, University of Tasmania, Hobart, TAS 7005, Australia; r.vaillancourt@utas.edu.au (R.E.V.); b.m.potts@utas.edu.au (B.M.P.); 2 CSIRO Land and Water, Hobart, TAS 7001, Australia; dale.worledge@csiro.au (D.W.); anthony.ogrady@csiro.au (A.P.O.)

**Keywords:** *Leptospermum*, genetic variation, heritability, provenance variation, flowering, precocity, growth, climate, manuka, plantation, common garden

## Abstract

*Leptospermum scoparium* is emerging as an economically important plant for the commercial production of mānuka honey and essential oils, both exhibiting unique antibacterial attributes. To support its domestication this is the first quantitative genetic study of variation for *L. scoparium* traits. It utilised plants from 200 open-pollinated families derived from 40 native populations, from across the species range in Tasmania, grown in a common garden field trial. The traits studied were survival, growth, and the flowering traits precocity, the timing of seasonal peak flowering, flowering duration, and flowering intensity. Significant genetic variation was evident at the population level for all traits studied and at the family level for three traits—growth, flowering precocity, and time to peak flowering. These three traits had moderate to high narrow-sense heritability estimates ranging from 0.27 to 0.69. For six of the traits studied, population differences were associated with climate attributes at the locations where seed was collected, suggesting adaptation to the local climate may have contributed to the observed population differentiation. Population level geographical trends suggest that genotypes to focus on for domestication originate from the eastern half of Tasmania for precociousness and the western half of Tasmania for earlier time to peak flowering and extended flowering duration.

## 1. Introduction

*Leptospermum scoparium* J.R. et G. Forst. is a member of the Myrtaceae family with a natural distribution limited to Australia (New South Wales, Victoria, Tasmania) and New Zealand. While the species is also cultivated for sale as ornamental shrubs [1,2], it is emerging as an economically important plant, with the main commercial products being mānuka honey made by *Apis mellifera* L. honey bees [3,4] and essential oils [5,6], both exhibiting unique antibacterial attributes. As such, the potential for the establishment of commercial *L. scoparium* plantations is receiving increasing interest [7]. Additionally, if integrated into existing farming systems as an agroforestry species, it may provide several benefits for the farming system. Plants can be successfully established on low-fertility soils, degraded lands, and steep hill country prone to erosion, which can help to mitigate soil loss and flood damage and reduce negative effects on water quality [8,9]. *Leptospermum scoparium* plantations can also reduce N-losses in silvopastural systems while producing a commercial crop [10]. 

Traits relevant to the successful establishment of *L. scoparium* plantations vary considerably in nature [11,12,13,14]. Successful development of plantations will likely require the identification and propagation of genotypes with high survival and fast growth, and for the production of honey, prolific flowering and elevated nectar production containing high concentrations of nectar bioactive compounds. The extent to which the variation in these traits has a genetic basis is poorly understood in this species, yet such knowledge is fundamental to its domestication. Phenotypic variation between and within natural populations may reflect genetic, environmental (both biotic and abiotic), or genotype by environment interaction components [15,16]. To determine if improvement of a species can be achieved through artificial selection and breeding requires an understanding of the quantitative genetic architecture of important traits, including the levels of genetic variation between and within populations as well as the correlations between traits [17]. Of particular relevance for the genetic improvement of populations are the relative levels of additive genetic variation of traits under selection, in other words their narrow-sense heritabilities, as well as the additive genetic correlations between traits [17]. As a rule, traits with greater heritability can be modified more easily by selection [18], but gains from multi-trait selection will in part depend upon the additive genetic correlations between them, which may act to constrain or facilitate the response to selection [19].

Despite the growing economic importance of *L. scoparium*, the quantitative genetic architecture of commercially relevant traits is poorly known, which hinders the development of breeding programs for the species. Genetic variance at the population level has been reported for some traits, along with some environmental influences on traits. In a discussion of ornamental cultivars, Dawson [13] notes that much of the variation in habit and flowering is likely to be genetically determined as these phenotypes are consistently maintained in cultivation. Ronghua et al. [20] found that for seedlings from a range of 17 New Zealand *L. scoparium* populations grown under uniform environmental conditions (glasshouse), the variation in form, leaf shape, flowering phenology, and age at first flowering had a significant genetic component. Furthermore, population variation was significantly correlated with geographic and climatic factors of the original collection site, such as latitude, distance from coast, as well as annual and winter temperatures. Using three cultivars grown in a glasshouse environment, Sheridan [14] reported varying levels of genetic control, environmental influence, and genotype by environment interaction influencing time to peak flowering, peak flower numbers, and flowering duration. Nickless et al. [21] found significant but complex interactions between cultivars and soils influencing plant growth, flowering, and nectar yield. However, of the research reported to date, we have found no studies that assess the family within population variation, nor that report the associated narrow-sense heritabilities and genetic correlations amongst *L. scoparium* traits. Notably, Thrimawithana et al. [22] has shown that the *L. scoparium* genome is highly syntenic with the *Eucalyptus* genome, a well-studied genus also from the same *Myrtaceae* subfamily (*Myrtoideae*), which provides a reference point for comparison.

To support the domestication of *L. scoparium* for the development of plantations, the current research aimed to investigate the genetic control of survival, growth (plant height), and flowering traits using plants from native open-pollinated *L. scoparium* seed lots collected from across the species range in Tasmania and grown in a common garden. The flowering traits studied included precocity, the timing of seasonal peak flowering, flowering duration, and flowering intensity. 

Survival, growth, and precocity are important factors influencing the successful establishment of plantations and reducing the time to commercial production. Precocious flowering also allows for faster generational turnover to increase the rate of incorporation of new genetic material into new commercial planting stock [23]. Other flowering traits such as timing of seasonal peak flowering, duration of flowering, and flowering intensity are also important considerations when maximising foraging by bees for the commercial production of bioactive honey [21]. 

The study specifically aimed to determine:

(i) population variation and its association with the climate of origin (i.e., home-site climate) of the seed lots;
(ii) variation among families within populations and associated narrow-sense heritabilities;
(iii) genetic correlations among traits to determine the constraints and synergies which may occur in multi-trait breeding; and
(iv) population and families that will provide genotypes with favourable traits for future breeding programs and commercial plantations.

## 2. Materials and Methods

### 2.1. Study Species

*Leptospermum scoparium* is typically a medium-sized shrub, but its habit may range from a semi-prostrate shrub (<1 m) to a tree up to 12 m tall [13,20]. It naturally occurs in a wide variety of habitats, including heaths, sedgelands, and woodlands, and in sites from sea level to approximately 1000 m in altitude [24]. The flowers are commonly white but can also range from pale pink to crimson and are 8–15 mm in diameter with up to five petals [1,25,26,27]. The main flowering season in the wild in Australia is from late September to early March [1], although some flowering can occur at any time of the year [28]. Individual flowers last from one to three weeks [26]. Individual plants produce two kinds of flowers: male flowers having functional stamens and non-functioning pistil, and hermaphrodite flowers in which both stamens and pistil are functional [29]. The extent and pattern of trait diversity seen in *L. scoparium* suggests that it exists as an open pollinating species of freely inter-fertile individuals [28]. Pollinators of *L. scoparium* include large tachinid and calliphorid flies, small Diptera, Coleoptera, Hymenoptera, moths, craneflies, and honeybees [27,30].

### 2.2. Sampling Sites and Seedling Production

**Figure 1:**
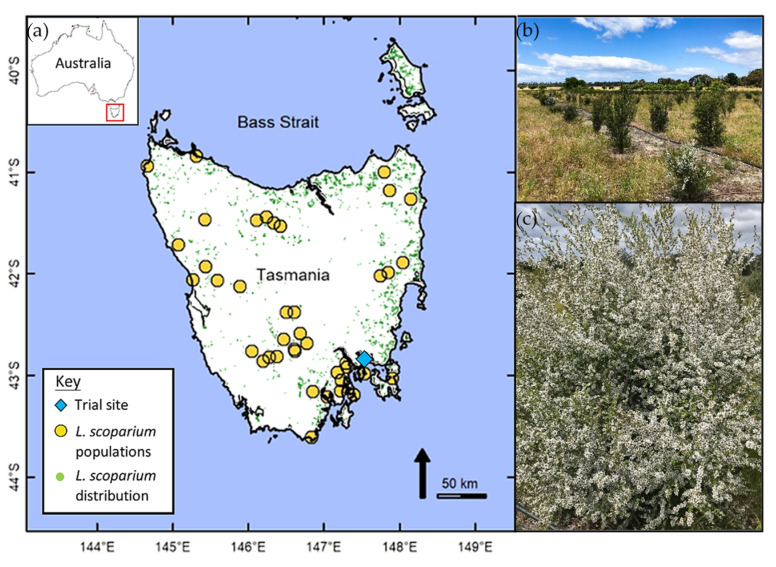
(**a**) Map of Tasmania showing trial site and population locations where open pollinated seed capsules of *Leptospermum scoparium* were collected. Green dots indicate recorded occurrences of *L. scoparium* obtained from Atlas of Living Australia [31], providing an indication of the species’ Tasmanian distribution. (**b**) Photograph of trial site plantation. (**c**) Photograph of *L. scoparium* in full flower.

Open-pollinated seed capsules were collected from 5 randomly selected plants in each of 40 populations (i.e., provenance) of *L. scoparium* (Permit number FL 18208) covering a wide geographic and altitudinal range of the species on the island of Tasmania (Figure 1, Table S1). The minimum distance between plants was at least two canopy heights. During sample collection, the latitude and longitude of each population was recorded. Seed capsules from each plant were air-dried in paper bags to allow seed release, and the seed was then stored in glass vials in family lots (i.e., all seed from one maternal plant). 

The open-pollinated seed lots were germinated in separate trays of forestry tubes (50 mm × 50 mm × 120 mm) at ambient temperature in the glasshouse facilities at CSIRO Land and Water, Hobart, Tasmania. As *L. scoparium* seed is very small, seed was sprinkled over the seed raising mix within forestry tubes, germinated, and then at four weeks thinned out such that only one seedling remained in each forestry tube. Seedlings were grouped in seedling trays by family, which were randomised with respect to population, and grown in a glasshouse for approximately 32 weeks. Seedlings were randomised into the experimental design just prior to planting at the trial site. The family identity of each plant, including source population, was maintained throughout.

### 2.3. Field Trial and Experimental Design

The common garden field trial is situated approximately 25 km east of Hobart (latitude 42.823175, longitude 147.510102, elevation 3 m; Figure 1). Climate at the planting site is classified as cool temperate maritime, with an average rainfall of approximately 500 mm per year and an annual pan evaporation of more 1300 mm per year. Mean daily maximum and minimum temperatures vary between 22.5 °C and 12.5 °C in summer and 12 °C and 4 °C in winter [32]. The soil is an Aeolian-derived sand 1–2 m deep over a heavy clay subsoil that promotes the development of perched water tables [32]. Prior use of the site involved a *Eucalyptus globulus* drought experiment from 2002 to 2009, then the site was left fallow until the *L. scoparium* common garden trial was established in mid-November 2017. 

The trial was established using a randomised block design with five replicate blocks, with families randomised within a replicate and represented by a single plot. Thus, each replicate contained a single plant from each of the 5 mother plants from each of the 40 populations, and overall, each family was represented by 5 plants. Plants were established with a 3 m × 3 m spacing and irrigated post establishment using inline dripper irrigation. Plants were irrigated with municipal water, with each plant receiving 4 litres of water 3 times a week from October to March each year. When plants died they were infilled with spare seedlings of the same family that had been retained from the original nursery stock. One population (CS) was replaced entirely with a new population (AS) at week 3 due to early complete mortality of that population. Data from replants were not used for analysis of the survival, growth, or precocity traits and were only used for other flowering phenology traits. 

### 2.4. Traits Measured

Data were collected on plant height, survival, and flowering over the first 39 months after trial establishment. This provided three summer flowering seasons (described here as years 1 to 3). Height, taken as the length of the main stem of a plant, was used as a measure of growth, and was measured every six months. Only the last height measurement after the third flowering season, at 34 months after plantation establishment, was used in the analyses. Survival was monitored every one to two months during the first 12 months, and thereafter recorded every one to three months. Flowering was assessed fortnightly during the peak summer flowering season of mid-November to mid-January and then monthly at other times. Flowering intensity was estimated using a modification of the Braun–Blanquet scale [33] to score the volume of the plant in flower from 0 to 7: with 0 representing no flowers on the plant; 1, ≤1% of the plant in flower; 2, >2% and ≤5% of the plant in flower; 3, >5% and ≤10% of the plant in flower; 4, >10% and ≤25% of the plant in flower; 5, >25% and ≤50% of the plant is in flower; 6, >50% and ≤75% of the plant in flower; and 7, >75% of the plant in flower. 

Flowering data were used to determine four flowering characteristics of potential interest to future breeding efforts for *L. scoparium*. Firstly, precocity (i.e., age to first flowering) was defined as the number of days to first flowering from plantation establishment. All plants monitored flowered within the experimental period. Secondly, time to peak flowering within a summer flowering season, was defined as the number of days after the 25th of September that the peak flowering score occurred. The third flowering trait was maximum flowering intensity, expressed as the highest flowering volume on the Braun–Blanquet scale within a flowering season. The fourth flowering trait was flowering duration, measured as the number of days that a plant flowered within a flowering season. The few plants that continued flowering past 5th February or started flowering after this date were excluded from analysis of this trait, as they were considered as flowering beyond the specific summer flowering season. As only 16.5% of plants flowered in the first summer after planting, analysis of all flowering traits except for precocity only utilised data from the summers of the second and third year. Flowering intensity, flowering duration, and time to peak flowering were set to missing values when a plant did not flower in a given year. 

### 2.5. Statistical Analysis

All statistical analysis was conducted using a combination of R version 4.0.0 [34] and ASReml-R Release 4 [35]. 

#### 2.5.1. Genetic Variances and Correlations

To estimate the proportion of phenotypic variance due to genetic effects associated with populations and families within populations, the following univariate linear model (model 1) was fitted to the data using either the maximum likelihood (R functions ‘lmer’ and ‘glmer’ in ‘lme4′ package) or the restricted maximum likelihood (ASReml-R) approaches:

*y* = µ + replicate + population + family (population) + ε model 1

where *y* is the observation, µ is the general mean, and ε is the residual. Replicate, population, and family (population) were set as random effects. For the trait survival a binomial model was fitted with a logit link function. All other models assumed a gaussian error distribution. Final models per trait were checked for linearity, homoscedasticity, and normality of residuals by plotting fitted against residual values and inspecting a histogram of residuals. One-tailed likelihood-ratio tests (LRT) of significance, conducted in R, were used to determine whether a variance component was significantly greater than zero. Due to the absence of literature on outcrossing rates in *Leptospermum* species, we assumed a mixed mating system and an average outcrossing rate of 70% for estimating narrow-sense heritability. This corresponds to the average outcrossing rate often assumed in quantitative genetic analyses of native open-pollinated seed lots of *Eucalyptus* [36,37], and in the absence of other information was done for comparative purposes. This assumption equates to assuming a coefficient of relationship within open-pollinated families of 0.4 (the reciprocal of which is 2.5). Narrow-sense heritability (h^2^_OP_) within populations was thus calculated as:

(1) $${h^{2}}_{\mathrm{OP}}=\frac{V_{A}}{\;V_{P}}=\frac{2.5\sigma^{2}{}_{f}}{\sigma^{2}{}_{f}+\sigma^{2}{}_{r}+\sigma^{2}{}_{res}}$$

where *V_A_* is the additive genetic variance, *V_P_* the total phenotypic variance, $\sigma^{2}{}_{f}$ the family within population level variance, $\sigma^{2}{}_{r}$ the replicate variance, and $\sigma^{2}{}_{res}$ the residual variance of the model. As the survival trait was modelled on the logit scale $\sigma^{2}{}_{res}$ was multiplied by $\frac{{𝜋}^{2}}{3}\approx 3.29$ [38].

Correlations between quantitative traits were estimated at the population and family within population levels, with the latter level estimating the additive genetic correlations between traits [39]. The family and population level estimates of trait–trait correlations were performed in ASReml-R version 4 by extending model 1 to the bivariate level. In these cases, the R matrix comprising the two variance components and correlations were estimated for the population, family (population), and replicate terms, with the ‘corh’ function, which allows heterogeneous variance component estimates. The residuals in this model were fitted with an unstructured covariance matrix. Variance components derived from the univariate analyses were used as starting values in these models. A two-tailed LRT, testing against the null model of covariance = 0 (‘diag’ covariance matrix for random effects), was sequentially used to calculate the significance of genetic correlations (i.e., family (population) and population correlations) at each level in the fitted model. Correlation values were only calculated where both traits showed significant (*p <* 0.05) variation at the level presented (population or family) in the univariate analysis.

#### 2.5.2. Population Spatial and Climate Associations

To visualize the geographic distribution of population level differences, population Best Linear Unbiased Predictions (BLUP) were spatially mapped using ‘maps’, ‘mapdata’, ‘mapproj’, ‘maptools’, and ‘rgdal’ packages in R [40,41,42,43,44]. The population level BLUPs were calculated for each trait from the univariate analyses using model 1 in R with the ‘lmer’ and ‘glmer’ functions from the ‘lme4′ package [45]. BLUPs were utilised instead of simple population means as they better predict genetic effects. 

To investigate past climate as a potential selection pressure driving the genetic differentiation at the population level, associations between quantitative trait population BLUPs and climate at the home site location of the wild original population were assessed. Latitude and longitude information was used to derive estimates of elevation and 35 climatic parameters (Table S2), representing a 30-year average from 1976 to 2005, for each population using the BIOCLIM prediction from ANUCLIM version 6.1 software [46]. To minimise redundancy and correlation between variables, principal components analysis (PCA) was used to reduce the 35 climate variables to two main dimensions, utilising the ‘prcomp’ built-in R function [34] and R package ‘FactoMineR’ [47]. The first two dimensions comprised a moisture gradient (PC1) and temperature gradient (PC2) that explained 52.7% and 16.7% of total climate variation in climate variables among the population locations, respectively (Figure S1). Pearson correlations of the population BLUPs with PC1, PC2, and elevation were calculated using ‘cor.test’ built-in R function [34]. 

## 3. Results

### 3.1. General Trait Observations

Table 1 provides trait codes and descriptive statistics for all *L. scoparium* plant traits studied. Of the plants originally planted, 61% survived during the life of the project. Nearly all mortality occurred within the first year, with a one-year survival probability of 62.5%. The average height of survivors was 129.4 cm after 1041 days. Mean age to first flowering was almost two years after trial establishment (709.2 days), and percentage of plants flowering by years 1, 2, and 3 were 16.5%, 78.4%, and 89.7% respectively. In comparing the year 2 and 3 summer flowering periods, year 2 had significantly shorter mean flowering duration of individual plants (paired t-test, df = 574, *p* < 0.001), significantly longer time to peak flowering of individual plants (paired t-test, df = 742, *p* < 0.001), and significantly greater maximum flowering intensity (paired t-test, df = 742, *p* < 0.001).

**Figure 2:**
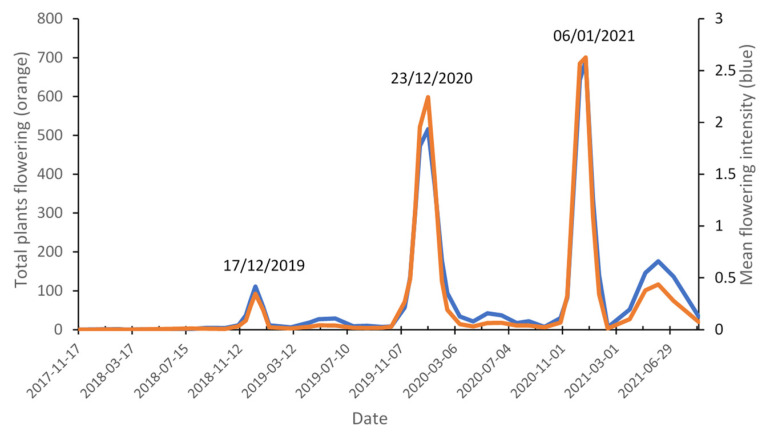
Total number of plants flowering and mean flowering intensity of *Leptospermum scoparium* over time in the first 3 years after plantation establishment. The main flowering peak each year occurs in summer (date indicated above each summer peak). Note that a secondary flowering peak also consistently formed in winter.

Figure 2 depicts the seasonal variation in both the total number of plants observed flowering and the mean flowering intensity of *L. scoparium* during the first 3 years after plantation establishment. The main flowering peak each year occurs in summer (November, December, January), and the flowering period is approximately 9 weeks for year 1 and 17 weeks for years 2 and 3. A secondary flowering peak also consistently formed in winter. Some plants flowered during winter but these did not represent unique families or populations and nearly all plants that flowered in the winter peak, also flowered in either the previous or following summer. 

### 3.2. Genetic Variation 

Highly significant (*p* < 0.001) genetic differentiation was found between populations for all traits, with differences among populations the dominant source of variation, particularly for the time to peak flowering (Table 2). Significant variation between families within populations was only detected for growth (i.e., height, *p* < 0.05), age to first flowering (*p* < 0.01), and time to peak flowering in both years 2 and 3 (*p* < 0.001; Table 2). For these traits within population narrow-sense heritability estimates (h^2^_OP_) ranged from 0.27 (growth) to 0.69 (peak flowering year 2). Variation among replicates within the field trial was significant for four traits (growth, age to first flowering, time to peak flowering year 2, and maximum flowering intensity year 3), but the estimated proportion of the total variation attributed to the replicate effect was minor compared with that due to genetic effects (i.e., population or family within population). 

**Figure 3:**
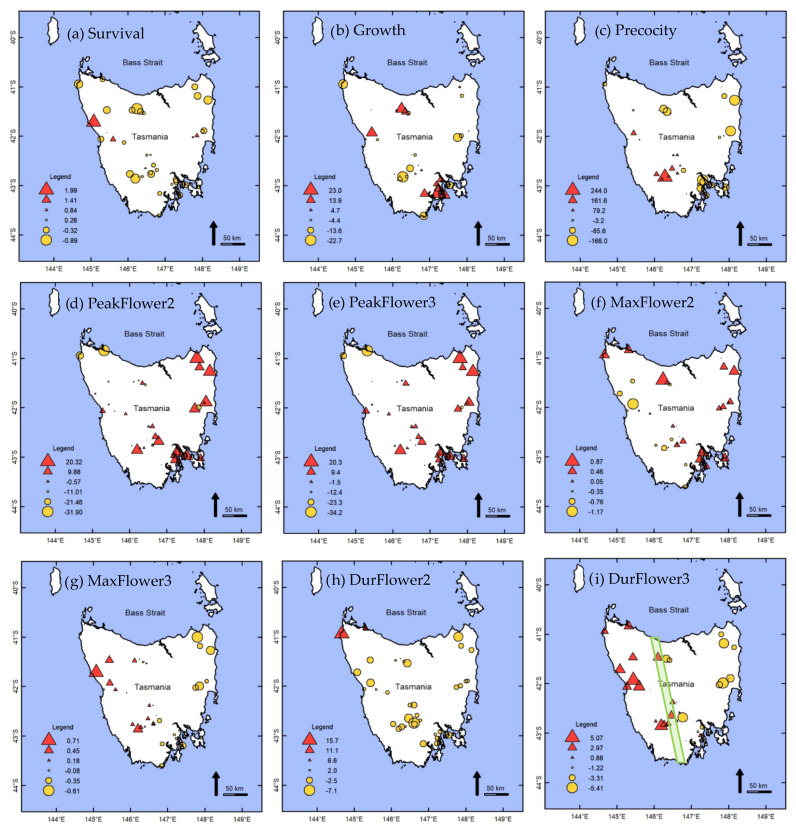
Geographic distribution of population BLUPs for traits of *Leptospermum scoparium* assessed in a common garden field trial. BLUPs are centered on the grand mean and the larger the circle or triangle, the greater the population BLUPs deviate above or below the grand mean, respectively. Tyler’s Line [48] shown in green in (**i**).

The geographic pattern of variation in the population level BLUPs across Tasmania is shown in Figure 3. For the survival and growth traits, no obvious geographical pattern is apparent. However, there were geographic patterns in the population BLUPs for the flowering traits across the island, mainly differentiating western and eastern populations. In the field trial, *L. scoparium* populations originating from the eastern half of Tasmania tended to flower at a younger age and more commonly took longer to reach peak flowering within a season (Figure 3). For maximum flowering intensity within a season, populations from eastern Tasmania or the north-west corner of Tasmania, tended to have a higher mean flowering score in year 2. However, this pattern was reversed in year 3, with populations from the western part of Tasmania exhibiting higher mean flowering scores. For the duration of flowering within a season, no obvious geographic trend occurred in year 2, whereas, within the year 3 season, plants from western Tasmanian populations flowered for a longer period. 

### 3.3. Genetic Correlations among Traits

Within population family-level correlations were only calculated between growth, precocity, and time to peak flowering years 2 and 3 (Table 2), as these were the only traits showing significant differentiation in the univariate analysis at the family-level (Table 3). However, all pairwise correlations among traits were estimated at the population level, and almost half were significantly different from zero (r = |0.48| to |0.98|, *p* < 0.05; Table 3). 

Survival was not found to be correlated with growth at the population level (r = −0.20, *p* = 0.21; data not shown in Table 3). Variation in precocity and flowering traits was not significantly correlated with plant growth at either the population or family levels (Table 3). Time to peak flowering was the trait under strongest genetic control (Table 2) and was highly positively correlated across seasons 2 and 3 at both the population (r = 0.98, *p <* 0.05) and family (r = 0.93, *p <* 0.001) levels. The population and family within population differences in flowering time are thus stable, despite the approximately 14-day difference in peak flowering dates between the two years. Populations from the western half of Tasmania that reached peak flowering earlier in the season (Figure 3) also tended to flower for a longer duration, but this correlation was only significant (r = −0.59, *p* < 0.001) in year 3 (Table 3). All flowering traits were one way or another correlated at the population level with flowering precocity. There was a weak trend for precocious populations to take longer to reach peak flowering in both year 2 (r = −0.52, *p* < 0.05) and year 3 (r = −0.40, *p* < 0.1), but this trend was not evident at the family within population level (Table 3). The sign of the population level correlation of precocity and both maximum flowering intensity and flowering duration was reversed between years 2 and 3. In year 2 precocious populations had greater maximum flowering intensity (r = −0.93, *p* < 0.001) and longer flowering duration (r = −0.57, *p* < 0.05), whereas in year 3 they exhibited a reduced maximum flowering intensity (r = 0.78, *p* < 0.001) and shorter flowering duration (r = 0.58, *p* < 0.05). These population level trends are further supported by the fact that maximum flowering intensity and flowering duration were positively correlated in both year 2 (r = 0.65, *p <* 0.01) and year 3 (r = 0.92, *p <* 0.001). As seen in the geographical trends in population level BLUPs (Figure 3), maximum flowering intensity is negatively correlated between years (r = −0.61, *p* < 0.05), and flowering duration is not correlated between years. 

### 3.4. Climate-Trait Associations 

Population level BLUPs were significantly correlated with the moisture gradient (PC1) at the home site of populations for five of the nine quantitative traits (Table 4). Plants from populations originating from wetter climates (positive values on PC1) first flowered at a later age (Precocity vs. PC1, r = 0.60, *p <* 0.001) and earlier in the season (PeakFlower2 vs. PC1, r = −0.37, *p <* 0.05). They flowered with less intensity in year 2 (MaxFlower2, r = −0.69, *p <* 0.001), but in year 3 flowered more intensely (MaxFlower3 r = 0.69, *p <* 0.001) and for longer duration (DurFlower3, r = 0.67, *p <* 0.001) than populations from drier areas. The direction of the correlation between the maximum flowering intensity BLUPs and PC1 was reversed between years 2 and 3, which concurs with the trend observed in the geographic representation of population level BLUPs (Figure 3). Only one trait, duration of flowering year 2, had a significant correlation with the temperature gradient (DurFlower2 vs. PC2, r = 0.47, *p <* 0.01) and elevation (r = −0.37, *p <* 0.05). 

## 4. Discussion

This is the first quantitative genetic study for *L. scoparium*, reporting narrow-sense heritability estimates and population variances for several agronomically important traits. The study used genetic material originating from a geographically broad collection on the island of Tasmania. Some traits were shown to be under high genetic control and thus amenable to genetic improvement. The study has identified populations that may provide high commercial value for the production of bio-active honey and essential oils by increasing precocity and flowering duration, as well as the ability to adjust peak flowering time to suit plantation production models. 

### 4.1. Plantation Phenotype Characteristics

Survival in the first year of planting was noticeably low at 62.5%, especially considering irrigation was present and weed control was ongoing. There are few reports of survival rates of *L. scoparium* in similar fenced and irrigated plantation settings, making comparison difficult. However, both Hamilton et al. [49] and Millner et al. [50] recorded survival rates of greater than 86% and 90%, respectively, for *L. scoparium* in New Zealand. In both cases it is unclear whether these plantings were fenced and irrigated. Again in New Zealand, Mardin and Lambie [51] found that survival after 4 years varied from 67% to 87%, despite the lack of fencing and irrigation. It is likely that the time of year for establishment is an important factor affecting survival rates, even in an irrigated setting. In our case the plantation was established in November just prior to a dry, warm summer. In contrast, both Hamilton et al. [49] and Millner et al. [50] established their plantings in the winter month of August, allowing the plants time to better establish roots before the summer season. Additionally, in 2018 the authors established an alternative fenced and irrigated plantation site at Douglas River, Tasmania, utilising similar seed stock as the Hobart study site, except seedlings were planted in the cooler, wetter month of June. The survival rate in the first year was 98.4%, supporting the above contention. 

For any *L. scoparium* commercial plantations focusing on either honey or essential oils, growth rates and time to flowering are important factors affecting when full production can occur. The average plant height at 1041 days (2 years and 10 months) was 129.4 cm. Saunders [52] reports expected growth rates of 40–50 cm per year in poor exposed non-irrigated soils in New Zealand, which would result in a total expected height after three years of 120–150 cm. Given that the study site was irrigated greater growth rates may have been expected. However, for most of the commercial uses of *L. scoparium* plant height is not as important as plant vigour, such as bushiness and health [52], and flowering onset and intensity. At the study site 89.7% of plants were flowering by the third summer. This accords with Saunders [52] in New Zealand, where commercial mānuka honey yield in plantations is expected to start in year 3 and reach maximum production by year 6. 

Mean flowering duration of individual plants differed significantly from 35.6 days in the much drier and marginally hotter second year to 46.1 days in the more moist and marginally cooler third year. A similar trend was found by Primack [53], in a study of 40 wild *L. scoparium* in New Zealand. Mean flowering duration for individual plants varied significantly from 17 days in a warm, dry summer to 34 days in a cool, damp summer in two consecutive years of observations in New Zealand. However, comparisons are made with caution as the study site was irrigated, whereas the wild population studied by Primack [53] was not. This difference in irrigation may be further highlighted by the fact that the study site had longer time to peak flowering and greater flowering intensity in the drier and marginally hotter second year, whereas Primack found these traits were reduced in the dry, warm summer. It is thus possible that dependent on site, irrigation may be important to maximise flowering intensity and total stand flowering time of *L. scoparium* plantations. 

While the mean flowering duration of individual plants ranged from 35.6 to 46.1 days (5–6.5 weeks), the total length of the stand summer flowering season extended much longer to approximately 17 weeks in years two and three. There was also a minor flowering peak in winter. The occurrence of winter flowering peaks of *L. scoparium* at the study site was expected given that Primack [53] found flowering occurring at all times of the year in New Zealand, and numerous ornamental cultivars are known to flower throughout winter [28]. Planted *L. scoparium* is also known to flower in winter in Israel [54]. In Tasmania, winter flowering appears to have poor genetic control, as the populations and families that flowered in winter were different across years. Additionally, it is unlikely to be of commercial significance as the Tasmanian winter weather is too cold for honey production and there is a limited number of plants flowering compared to the summer flowering season. 

### 4.2. Genetic Variation Underlying Quantitative Traits

Significant genetic differentiation was evident at the population level for all traits studied, and at the family within population level for three traits. It should however be noted that this study was conducted at only one site, and thus the relative importance of genotype x environment interactions, as for example reported by Sheridan [14] and Nickless et al. [21] for time to peak flowering, peak flower numbers, and flowering duration, is unclear. 

#### 4.2.1. Growth

Significant genetic variation was found for growth at both the population level and family level, with a moderate heritability estimate obtained (h^2^_OP_) of 0.27. Genetic control of growth has been previously reported for *L. scoparium* in New Zealand at the population level [21], but this is the first estimate of narrow-sense heritability for growth in the species. Similar moderate narrow-sense heritability values for growth have been estimated from families derived from wild open-pollinated seed collection of other Myrtaceae such as *Eucalyptus globulus* (0.2: [55]) and *E. cladocalyx* (0.21 to 0.37: [56]). The significant genetic variation and heritability found for growth was not biased by the significant variation in survival as these two traits were not correlated. However, it should be noted that heritability estimates from open-pollinated families may be affected by outcrossing rate and its variation, especially for traits that can be subject to inbreeding depression [37,57]. In *E. globulus*, heritability estimates for growth are inflated when measured from open-pollinated progenies due to variation in inbreeding depression [58,59]. However, we do not know whether this is also the case in *L. scoparium*.

Population level BLUPs for growth did not exhibit an obvious geographical trend and were unrelated to home-site climate, making it difficult to understand what may be driving variation among populations, and to ultimately predict the locations to focus for establishing breeding populations. There appears to be a strong cluster of populations with higher growth performance near the study side, which could signal local adaptation to environmental factors which are not climate related. However, this is not the only cluster of high-performance populations, indicating that alternative explanations not studied here may have played a past role in driving the genetic variation observed, such as population variation in outcrossing rate and inbreeding depression, as mentioned above. This may similarly be an explanation for the population variation in survival. 

#### 4.2.2. Precocity

Flowering precocity was found to be under strong genetic control, with significant genetic variation at both the population and family level, and the narrow-sense heritability (h^2^_OP_) estimated to be 0.31. Significant genetic variation in precocity, at both the population and family levels, have been reported for several eucalypt species. Moderate narrow-sense heritability values have been recorded for *E. globulus* (0.18 to 0.24: [60,61]) and *E. risdonii*—*E. tenuiramis* complex (0.31 to 0.41: [62]), whereas higher values have been noted for *E. cladocalyx* (0.48 to 0.57: [56]). 

Importantly, precocity and growth were not correlated, indicating that these traits are under independent genetic control. Thus, breeding selection for early flowering would not impact early growth. The lack of correlation between growth and precocity supports the findings of Nickless et al. [21] for *L. scoparium*. These traits were also found to be independent in *E. globulus* from Tasmania [60,61], as was vegetative phase change and first flowering in *E. risdonii*—*E. tenuiramis* complex [62] and the crop species *Zea mays L.* sweet corn [63], suggesting that this independence may be widespread in plants. 

In terms of trait–environment associations, at the population level variation in precocity in the common-garden was highly correlated to the provenance home-site moisture gradient, whereas growth was not, highlighting a likely difference in selection forces acting on these traits. The geographical pattern of population level BLUPs for precocity across Tasmania suggest past drivers of selection affecting this trait that tend to coincide with the west–east provincial division along Tyler’s Line [48], such as rainfall and/or geology/soil (Figure 3i). Tyler’s Line runs from just south-east of the north-western corner of Tasmania, and continues south, cutting roughly down the centre of the island [48]. The two regions possess major differences in climate, geology, and vegetation, with the west having higher mean rainfall (2000–3500 mm/year) and poor acidic soil, while the east has a lower mean rainfall (800–2000 mm/year) but slightly more fertile soil [48]. The strong correlation found between the population level variation at the common garden site and a climate moisture gradient at the population home site, indicates that local adaptation to climate is a possible driver of the genetic variation in precocity amongst populations. The geographical trend indicates that populations from the drier sites in eastern Tasmania tended to reach flowering age quicker. This may be evidence of a constitutive adaptation to home-site water availability. For example, heritable earlier flowering has been found in provenances from drier sites for *E. occidentalis* [64] and hotter sites for *E. globulus* [65]. This may also point to an association of early flowering with greater fire frequencies as suggested by Potts [66] for a mallee population of *E. gunnii*. An alternative hypothesis for the observed correlation between the climate moisture gradient and precocity is that it is a plastic response to stress [67,68]. Stress is known to induce precocious flowering in *Citrus latifolia* [69], Arabidopsis [70], the perennial woody tree *Sapium sebiferum* [71], and some ephemeral native plants and cereal crops such as wheat [72]. However, this is unlikely to be the case here as there are no parallel trends in survival or growth that suggest the eastern populations are under stress in the trial. Additionally, the trial site is located in eastern Tasmania so it is unlikely that the eastern populations are more maladapted to this climate than the western populations.

#### 4.2.3. Flowering Intensity and Duration

Both flowering intensity and flowering duration exhibited highly significant genetic variation at the population level, but not at the family level. These two traits were also highly correlated within both years 2 and 3. Published literature appears limited on the relationship between these two traits, however flowering duration and number of flowers have been reported as positively correlated for the Chinese endemic species *Eomecon chionantha* [73]. While the genetic correlation between these traits may be the product of genetic linkage [74] it most likely represents pleiotropic gene effects, in this case arising from a type of allometric effect where plants with more flowers sustain longer flowering duration. In year 2, both increased flowering intensity and extended flowering duration are also correlated with precocious flowering at the population level, suggesting that the populations from the eastern half of Tasmania have a greater propensity for precocious and heavier flowering. However, the reverse trend was evident the following year with the more precocious and initially heavier flowering populations having lower maximum flowering intensity and flowering duration in year 3. With respect to flowering duration, geographical trends of population level BLUPs only become apparent by year 3, indicating that complete genetic expression of this trait may be delayed. Regarding maximum flowering intensity, the reversal in the population level trend between years may be due to several potential factors. The higher flowering intensity in populations from the eastern half of Tasmania in year 2 may be driven by that area having more precocious plants. This may then have been followed by a change in reproductive resource allocation in year 3 to vegetative growth, and/or a reduction in available resources due to the cost of heavy flowering in year 2. *Eucalyptus camaldulensis* has been shown to flower more intensely every second year and with some dependence on the previous year flowering intensity [75]. Flowering patterns for a range of other plant species are known to change dramatically from year to year, both at the species and community levels [76]. Alternatively, the population level trends observed may be due to a differing climate adaptation response at the common garden site. Flower production is known to be positively correlated with flowering season rainfall for eucalypts [77,78], and this study has shown population level BLUPs for flowering intensity to be highly correlated with the moisture gradient at the population home site, but the trends were reversed between years 2 and 3. This could reflect differential adaptation to the prevailing rainfall regime at the trial site. Year 2 flowering season at the common garden site was drier and marginally hotter, which may suit populations originating from the drier eastern half of Tasmania, whereas year 3 was wetter and cooler, potentially suiting populations originating from the wetter western half of Tasmania. To confirm which of these driving forces are influencing the observed pattern for flowering intensity additional data collection and analysis is required beyond year 3. 

#### 4.2.4. Time to Peak Flowering

Of all traits studied, time to peak flowering was under the strongest genetic control at both the population and family level, with the highest narrow-sense heritability estimates (h^2^_OP_) of 0.62 to 0.69. Similarly very high heritability values (>0.64) have been reported by Potts et al. [79] for start, peak, and end of flowering in *E. globulus*. The remarkable year to year stability in time to peak flowering across seasons, both at the population level (r = 0.98) and family level (r = 0.93), accords with findings by Primack (1980) for seasonal phenotypic trends observed in a single wild population of New Zealand *L. scoparium* and a study by O’Brien and Calder [80] regarding 20 plants of both *L. myrsinoides* and *L. continentale* from each of three populations. Such strong genetic control over timing of flowering may be a result of the advantage it provides populations to produce an extensive floral display to attract pollinators and to increase the chance of cross pollination [81]. 

Time to peak flowering was not correlated with growth, demonstrating independent genetic control of these traits. Time to peak flowering and precocity were not correlated at the family level, indicating the two traits are under independent genetic control [82]. However, the fact that both precocity and peak flowering are negatively correlated at the population level, and in year 2 both were correlated with moisture gradient at the home site, implies there may be some similar selection forces that acted on these traits, albeit acting on different genes. 

The geographical representation of population level BLUPs indicated that by year 3 the populations originating from the western half of Tasmania reached peak flowering earlier, flowered with greater intensities, and for longer durations. While these trends were consistent with population level correlations for year 3, the relationship between time to peak flowering and flowering intensity was not statistically significant. In Primack’s study [53] of a wild *L. scoparium* population in New Zealand over a two-year period no correlations were found between peak flowering time and flowering intensity or duration, whereas a significant correlation was found between peak flowering time and fruit set in one year. However, the study only focused on 40 individual plants from a single population in the wild. Heat sum accumulated during a flowering season is often a major driver of flowering time in plants [83]. Thus, a possible explanation for the populations from the western half of Tasmania reaching peak flowering earlier is that because it is colder in the west coast, the plants there have evolved to require less heat sum to initiate flower buds or develop these buds through to flowering, so their time to peak flowering is shorter in the plantation.

## 5. Conclusions

Significant genetic differentiation was evident at the population level for all traits studied, and at the family level for three traits, suggesting a strong capacity for genetic improvement of *L. scoparium*. Thus, commercially relevant gains can be achieved in survival, growth, and flowering traits through provenance and family selection for breeding and deployment into plantations. As growth and precocity were found to have independent genetic control, genotype selection based on precocity alone would generally not impact early growth. Selection for precocious plants will not only assist with faster generational turnover in the breeding efforts but may also aid in reducing time to commercial production. The fact that flowering intensity and duration were found to be positively correlated is advantageous from a breeding perspective as increased flowering intensity and extended flowering period will strengthen production in a commercial plantation setting. Time to peak flowering was under the strongest genetic control and remarkably stable between years, meaning that very reliable control can be achieved in the relative timing of flowering of *L. scoparium* genotypes in a commercial plantation setting. This may assist with plantation design by either broadening or tightening the total plantation flowering time, depending on the purpose of the commercial application. With the current information available, population level geographical trends suggest that genotypes to focus on originate from the eastern half of Tasmania for precociousness, and the western half of Tasmania for earlier time to peak flowering and extended flowering duration. 

## Figures and Tables

**Table 1:** Trait codes and descriptive statistics for all *Leptospermum scoparium* plant traits studied. The survival, height, and precocity data are only for the original planting, whereas the flowering peak and durational data included replants.

Trait Description	Trait Code	Units	n	Min	Max	Mean (SD)
Survival	Survival	0–1	975	0	1	0.61
Height	Height	cm	587	15	235	129.4 (36.9)
Age to first flowering	Precocity	days	594	91	1160	709.2 (199.5)
Time to peak flowering year 2	PeakFlower2	days	766	51	118	94.3 (15.4)
Time to peak flowering year 3	PeakFlower3	days	877	44	118	85.2 (13.4)
Maximum flowering intensity year 2	MaxFlower2	0–6	766	1	7	5.4 (1.5)
Maximum flowering intensity year 3	MaxFlower3	0–6	877	1	7	4.8 (1.6)
Duration of flowering year 2	DurFlower2	days	589	10	95	35.6 (12.5)
Duration of flowering year 3	DurFlower3	days	871	9.5	97.5	43.1 (14.1)

**Table 2:** The proportion of the total variation attributable to random replicate, population, and family within population effects, their significance, and within population narrow-sense heritability (h^2^_OP_) for growth and reproductive traits of *Leptospermum scoparium* grown in a common garden (standard errors are indicated in brackets).

Trait Code	Variance Proportion (SE) and Significance			h^2^_OP_ (SE)
	Replicate	Between Populations	Family within Population	
Survival	0.01 (0.02) ^ns^	0.29 (0.08) ***	0.11(0.07) ^ns^	0.13 (0.09)
Growth	0.04 (0.03) ***	0.14 (0.05) ***	0.09 (0.04) *	0.27 (0.12)
Precocity	0.03 (0.03) ***	0.26 (0.06) ***	0.09 (0.04) **	0.31 (0.12)
PeakFlower2	0.01 (0.01) **	0.71 (0.05) ***	0.08 (0.02) ***	0.69 (0.11)
PeakFlower3	0.00 (0.00) ^ns^	0.66 (0.06) ***	0.08 (0.02) ***	0.62 (0.10)
MaxFlower2	0.00 (0.00) ^ns^	0.13 (0.04) ***	0.04 (0.03) ^ns^	0.10 (0.09)
MaxFlower3	0.03 (0.02) ***	0.06 (0.02) ***	0.03 (0.03) ^ns^	0.07 (0.07)
DurFlower2	0.00 (0.00) ^ns^	0.17 (0.04) ***	0.02 (0.03) ^ns^	0.05 (0.10)
DurFlower3	0.01 (0.01) ^ns^	0.09 (0.03) ***	0.04 (0.03) ^ns^	0.11 (0.08)

* *p* < 0.05, ** *p* < 0.01, *** *p* < 0.001, ns = not significant, derived using a one-tailed.

**Table 3:** Population level and family-level genetic correlation and standard error between *Leptospermum scoparium* traits. Trait codes are provided in Table 1. Correlation results were only calculated where both traits showed significant variation at the level presented (population or family) in the univariate analysis.

Trait Code	Level	Plant Trait						
		Precocity	PeakFlower2	PeakFlower3	MaxFlower2	MaxFlower3	DurFlower2	DurFlower3
Growth	Population	−0.31 (0.20) ns	0.22 (0.20) ns	0.25 (0.19) ns	0.20 (0.23) ns	−0.01 (0.28) ns	−0.01 (0.24) ns	−0.03 (0.25) ns
	Family	−0.01 (0.33) ns	−0.03 (0.21) ns	0.07 (0.21) ns	na	na	na	na
Precocity	Population		−0.52 (0.14) **	−0.40 (0.16) ^†^	−0.93 (0.07) ***	0.78 (0.17) **	−0.57 (0.15) *	0.58 (0.18) *
	Family		−0.01 (0.20) ns	−0.01 (0.19) ns	na	na	na	na
PeakFlower2	Population			0.98 (0.01) ***	0.36 (0.17) ns	−0.48 (0.18) *	−0.23 (0.19) ns	−0.61 (0.14) **
	Family			0.93 (0.07) ***	na	na	na	na
PeakFlower3	Population				0.20 (0.19) ns	−0.40 (0.20) ns	−0.42 (0.17) ns	−0.59 (0.15) **
	Family				na	na	na	na
MaxFlower2	Population					−0.61 (0.18) *	0.65 (0.14) **	−0.51 (0.19) *
	Family					na	na	na
MaxFlower3	Population						−0.26 (0.25) ns	0.92 (0.10) ***
	Family						na	na
DurFlower2	Population							0.15 (0.24) ns
	Family							na

^†^*p* < 0.1, * *p* < 0.05, ** *p* < 0.01, *** *p* < 0.001, ns = not significant.

**Table 4:** Correlation of population level BLUPs with PC1, PC2, and elevation, for *Leptospermum scoparium* traits.

Trait Description	Moisture Gradient (PC1) ^1^	Temperature Gradient (PC2) ^2^	Elevation
Survival	0.18 ^ns^	−0.27 ^ns^	0.22 ^ns^
Growth	−0.04 ^ns^	0.08 ^ns^	−0.24 ^ns^
Precocity	0.60 ***	−0.17 ^ns^	0.23 ^ns^
PeakFlower2	−0.37 *	−0.08 ^ns^	−0.07 ^ns^
PeakFlower3	−0.24 ^ns^	−0.09 ^ns^	−0.02 ^ns^
MaxFlower2	−0.69 ***	0.06 ^ns^	−0.20 ^ns^
MaxFlower3	0.69 ***	−0.18 ^ns^	0.30 ^ns^
DurFlower2	−0.23 ^ns^	0.47 **	−0.37 *
DurFlower3	0.67 ***	0.28 ^ns^	−0.04 ^ns^

* *p* < 0.05, ** *p* < 0.01, *** *p* < 0.001, ns = not significant. ^1^ Increasing values on PC1 are associated with increasing precipitation and moisture index variables and decreasing radiation and moisture index seasonality. ^2^ Increasing values along PC2 are associated with increases in temperature variables and decreased temperature range and seasonality variables.

## Data Availability

The data presented in this study are available on request from the corresponding author.

## Supplementary Materials

The following supporting information can be downloaded at: https://www.mdpi.com/article/10.3390/plants11081029/s1: Table S1. Population locations and arithmetic means for traits. Table S2. 35 BIOCLIM climatic parameters from ANUCLIM version 6.1 software [46]. Figure S1. Principal component map showing dimensions PC1 and PC2 for 35 Bioclim variables (see Table S2 for variable codes and names) at the 40 population locations.
