# Supplementary material for: Genetic Variation in Flowering Traits of Tasmanian Leptospermum scoparium and Association with Provenance Home Site Climatic Factors
Source: Plants (Basel). 2022 Apr 10;11(8):1029. doi: 10.3390/plants11081029 (PMC9029099; doi:10.3390/plants11081029)
Supplement: Supplementary file 1 [file plants-11-01029-s001.zip › plants-1632294-supplementary.pdf]

Supplementary Material

Table S1. Population locations and arithmetic means for traits

Population	Latitude	Longitude	Survival	Precocity	Growth	PeakFlower2	PeakFlower3	MaxFlower2	MaxFlower3	DurFlower2	DurFlower3
**ac**	-41.5349	146.4300	0.6	797.47	114.13	80.53	80.07	4.60	5.06	13.93	27.28
**as**	-42.1268	145.8892				82.97	79.22	4.40	6.09	17.70	32.11
**ca**	-41.0031	147.8040	0.72	761.89	134.00	114.55	106.53	5.13	3.70	20.25	18.25
**cr**	-42.3790	146.6077	0.52	819.23	117.62	96.53	87.64	5.00	4.96	21.06	27.10
**cr2**	-41.4462	146.2387	0.88	586.55	154.86	90.03	81.55	6.44	4.96	19.68	29.98
**cs**	-41.7167	145.0764	0								
**el**	-42.5873	146.6919	0.64	721.56	126.81	100.58	89.18	5.14	4.96	18.38	26.90
**en**	-43.0316	147.9033	0.64	599.13	130.93	102.53	94.36	6.00	4.52	20.95	25.00
**er**	-42.6503	146.4684	0.36	865.00	100.00	87.21	79.24	4.55	5.30	15.64	32.15
**flo**	-42.7639	146.6162	0.76	758.05	122.42	80.99	77.86	5.65	5.16	15.13	32.24
**fp**	-42.8135	146.3855	0.56	822.00	119.43	92.93	83.29	5.16	5.09	17.53	31.20
**gf**	-41.5049	146.3431	0.72	573.00	145.94	96.01	90.20	5.46	4.67	17.10	20.54
**gm**	-42.0662	145.5958	0.24	816.67	115.17	85.18	79.44	4.83	5.36	18.00	33.07
**go**	-41.2700	148.1544	0.8	524.25	128.15	112.43	104.04	6.08	4.00	22.17	17.63
**gv**	-43.1637	146.8591	0.64	739.94	150.13	85.82	79.06	4.68	4.43	17.21	29.65
**hd**	-42.0643	145.2715	0.72	782.83	132.65	98.43	92.66	4.71	4.70	22.58	26.33
**ju**	-42.7457	146.6140	0.64	810.94	128.60	83.37	76.95	4.83	5.29	14.35	32.28
**kl**	-42.8804	147.2921	0.72	522.28	144.61	111.21	96.36	5.87	4.79	19.36	20.63
**lg**	-42.9719	147.1818	0.56	708.14	142.57	99.67	86.18	5.14	4.58	19.18	29.10
**ll**	-42.0226	147.7494	0.52	697.62	100.92	108.10	92.86	5.67	4.26	16.22	22.02
**lm**	-43.2120	147.0527	0.44	814.82	138.09	95.91	84.67	5.52	4.50	19.47	24.56
**lp2**	-42.7667	146.0524	0.76	852.00	130.63	88.17	81.56	4.63	5.26	15.32	29.24
**md**	-41.9338	145.4401	0.44	882.18	157.82	83.02	81.40	3.30	5.53	13.45	35.24
**mf**	-41.9910	147.8574	0.28	795.86	115.29	73.97	69.67	5.79	3.96	17.57	28.74
**mf2**	-43.1961	147.3967	0.68	637.24	153.50	94.51	83.46	5.79	4.13	20.43	28.33
**mg**	-43.0542	147.2339	0.52	595.92	151.08	106.45	91.79	6.00	4.62	17.92	27.67

**mn**	-42.9180	147.3152	0.72	587.22	123.56	109.84	96.13	6.18	4.88	27.40	25.96
**mo**	-41.1824	147.8758	0.76	651.53	116.44	105.28	92.60	5.83	4.30	18.26	20.11
**nc**	-42.9837	147.5365	0.72	628.61	105.22	109.90	97.40	5.38	4.57	20.61	21.46
**oc**	-41.8903	148.0472	0.72	527.83	126.06	110.98	98.65	5.75	4.43	17.33	19.28
**pb**	-40.8417	145.3148	0.68	716.18	124.12	60.62	49.50	5.96	4.88	27.17	27.62
**pr**	-43.1527	147.2280	0.52	722.38	163.46	86.57	81.87	5.30	4.91	17.53	26.36
**sec**	-43.6075	146.8391	0.6	725.13	103.27	84.58	77.43	4.47	4.32	18.58	31.50
**sr**	-42.8610	146.1992	0.84	815.90	142.10	109.43	100.24	5.10	5.70	22.63	24.77
**wa**	-42.3806	146.5078	0.56	813.29	129.14	92.33	84.53	5.45	5.17	17.29	29.19
**wd**	-42.8227	146.2778	0.4	1049.20	91.00	82.60	79.07	4.00	5.32	15.13	33.47
**wm**	-41.4771	146.1090	0.72	729.83	136.56	84.91	80.02	5.05	5.32	17.83	33.41
**wp**	-40.9419	144.6682	0.8	668.25	99.84	68.56	60.63	6.04	4.71	30.44	34.75
**wt**	-41.4688	145.4266	0.76	720.47	129.11	83.62	79.03	4.52	5.57	16.96	31.33
**ww**	-42.6855	146.7779	0.6	653.93	135.47	107.40	94.94	5.73	4.36	20.92	19.18

Table S2. 35 BIOCLIM climatic parameters from ANUCLIM version 6.1 software (Xu and Hutchinson 2010).

Code	BIOCLIM Variable
1	Annual Mean Temperature
2	Mean Diurnal Range(Mean(period max-min))
3	Isothermality 2/7
4	Temperature Seasonality (C of V)
5	Max Temperature of Warmest Period
6	Min Temperature of Coldest Period
7	Temperature Annual Range (5-6)
8	Mean Temperature of Wettest Quarter
9	Mean Temperature of Driest Quarter
10	Mean Temperature of Warmest Quarter
11	Mean Temperature of Coldest Quarter
12	Annual Precipitation
13	Precipitation of Wettest Period
14	Precipitation of Driest Period
15	Precipitation Seasonality(C of V)
16	Precipitation of Wettest Quarter
17	Precipitation of Driest Quarter
18	Precipitation of Warmest Quarter
19	Precipitation of Coldest Quarter
20	Annual Mean Radiation
21	Highest Period Radiation
22	Lowest Period Radiation
23	Radiation Seasonality (Cof V)
24	Radiation of Wettest Quarter
25	Radiation of Driest Quarter
26	Radiation of Warmest Quarter
27	Radiation of Coldest Quarter
28	Annual Mean Moisture Index
29	Highest Period Moisture Index
30	Lowest Period Moisture Index
31	Moisture Index Seasonality (C of V)
32	Mean Moisture Index of High Qtr.MI
33	Mean Moisture Index of Low Qtr.MI
34	Mean Moisture Index of Warm Qtr.MI
35	Mean Moisture Index of Cold Qtr.MI

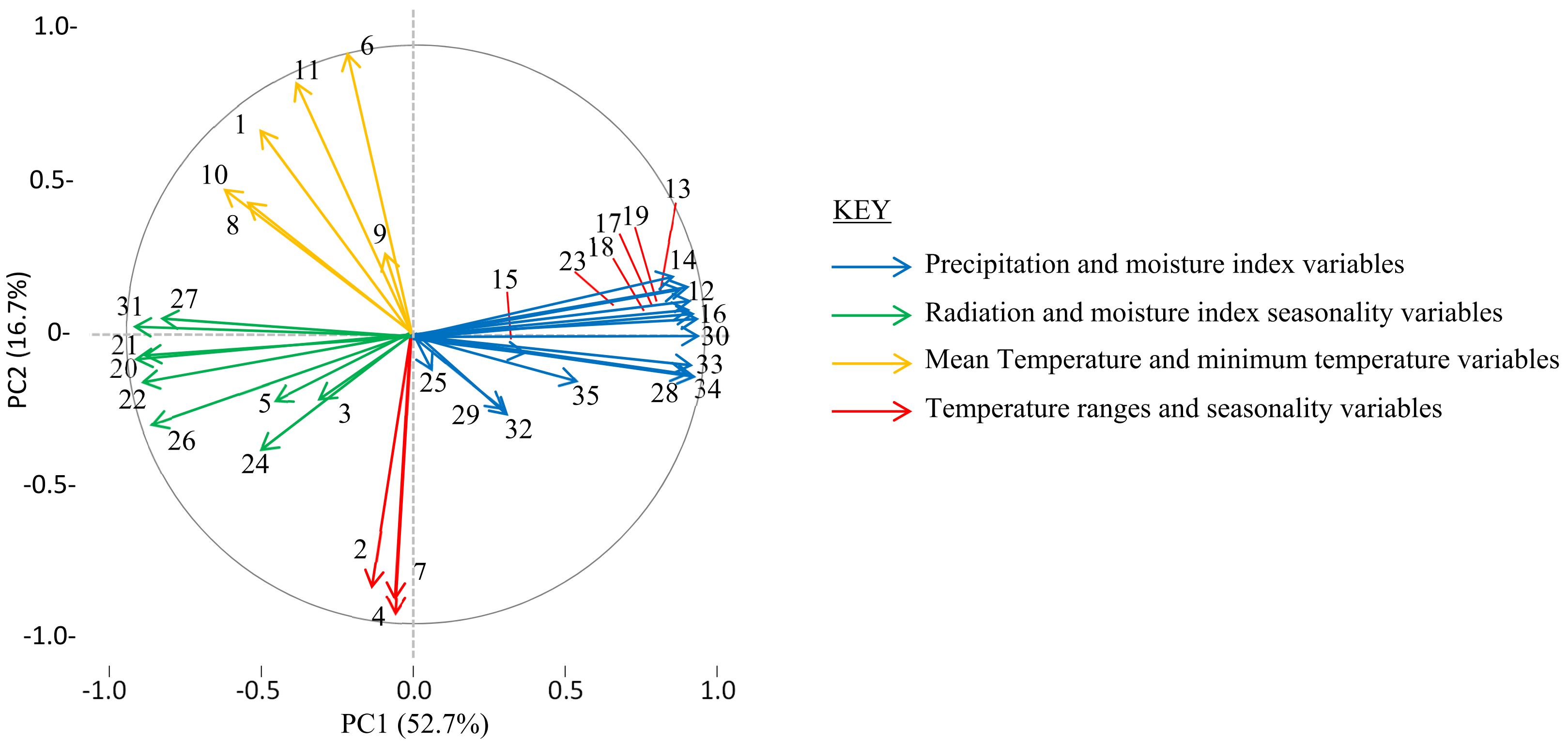

Supplementary Figure S1. Principal component map showing dimensions PC1 and PC2 for 35 Bioclim variables (see Table S2 for variable codes and names) at the 40 population locations.
